# Pyromania—A Paper Read before the Erie County Medical Society at the Annual Meeting, January 12, 1869

**Published:** 1869-02

**Authors:** William C. Phelps


BUFFALO

Medical and Surgical Journal.

VOL. VIII. FEBRUARY, 1869. No. 7.

### Original Communications.

ART. I.—*Pyromania—A Paper read before the Erie County Medical Society at the Annual Meeting, January* 12, 1869. By William C. Phelps, M. D.

Having had my attention directed to this form of mental disease by a legal proceeding, I thought that I could not better fulfill the duty which has been imposed upon me, which must be but poorly discharged at best, than by giving what I have been able to glean from the writings of alienist physicians concerning this strange and rare form of mental disease.

Pyromania—the monomania incendiare of Esquirol—is ranked as a disease or perverted action of the moral faculties, and comes under the division of the monomanias without delusion of most writers—the moral insanity of Pricard—the emotional insanity of Tuke. It is difficult to select a name which will satisfactorily designate a mental disorder, for the reason that so little is known as to the nature of insanity in general. Did we perfectly understand the faculties of the mind we could easily arrange a satisfactory nomenclature, according as one or more of them are affected; or did we perfectly understand the physiology of the organ of the mind, we could then name the disease according to the particular part affected, but in the absence of both these data we are obliged to select names which will express most plainly the prominent symptoms. Hence the name pyromania which has been given this disease by Marc, the most prominent symptoms being the uncontrollable desire to fire buildings of every description, generally without any feelings of revenge or malice. In order that its nature may be better understood, I will briefly notice the twofold division of the mind.

Metaphysicians in all ages have very generally agreed that the manifestations of the mind are divided into two great classes—the intellectual and the moral or affective. Plato, as quoted by Tuke, tells us that he distinguishes two principal faculties; that of feeling and that of thinking. "To feel," he says, "is to be affected by an external impression; to think is to operate on our own ideas." Modern writers have preserved and enforced this distinction. Reed, in his analysis of the mental faculties, comprises two divisions—the understanding and the will. Brown and Welsh, in their work, divide the affections of the mind into two classes, the intellectual and the emotional; and Martin Payne of our own country, in his Institutes of Medicine, remarks, that "our emotions differ so manifestly from our intellectual states of being by that peculiar vividness of feeling which every one understands, though it may be impossible to embody it in any verbal definition, that it is not a little singular that one should be confounded with the other by any who have simply remembered and compared, and have also loved or hated, desired or feared."

Phrenological and some recent writers still further sub-divide the emotions into the higher sentiments and the propensities. This division is desirable and convenient when analyzing diseases of the emotions, being among the few valuable ideas left us from the wreck of phrenology, which once promised to teach us the correct physiology of the brain, and make straight the crooked ways of our mental science.

The foregoing view—that of the duality of the mind—is the one most generally accepted, as it is sustained by facts obtained from observations of its faculties in health and disease. The opposite opinion, however, that of the solidarity of the mental faculties, is held by those of acknowledged eminence in this department of medical research. They throw aside this metaphysical view of the mind and insist that it is a unit, and that when diseased all its faculties are diseased together, though some will be more prominently developed than others. This point was strongly insisted on by some speakers in the discussions which occurred at a recent meeting of the Medico-Psychological Society in Paris. But a reviewer of that discussion in the *American Journal of the Medical Sciences*, remarks that "if this were a question of pure metaphysics, there might be some reason for the belief in question, but it must be considered that the brain is not a unit in the sense in which that term is applied to the mind, but a heterogeneous organ, the different parts of which all analogy teaches us exercise different functions in the mental economy."

It is this second division of the mental faculties then—the sentiments and propensities which are primarily affected in pyromania, belonging as it does to that group of mental diseases which includes homicidal and suicidal mania, kleptomania, dipsomania, etc., called emotional or moral insanity, it being, if I may be allowed the simile, but one of the windows at which reasoning madness shows itself. We shall, therefore, consider the general characteristics of the group in connection with the special derangement, pyromania.

The mind is so complex in its manifestations that it is somewhat difficult to determine the point at which health ceases and disease begins. But this difficulty which exists in all departments of medical knowledge is more seeming than real, for if we take the natural and habitual character of the patient, which is the best test of disease, as the standard of comparison, and consider all unnatural manifestations of the mind as due to disease of its organ, either functional or organic, in practice the difficulty in pronouncing between the sane and insane, will be greatly lessened, especially in criminal cases; and I will remark, though it has no necessary connection with the present subject, that the fallacy of "momentary insanity" which was recently allowed by a court of justice in this State, is plainly to be seen from this stand point, for no physician would for a moment assert that the brain could become so seriously and suddenly diseased for a few seconds of time as to cause homicidal mania, and as suddenly return again to a state of health. Disease, says Chomel, "is a notable disorder, affecting more or less of the constituent parts of the living organism, as regards either their material constitution or the exercise of their functions." And this definition is applicable to the brain as to any other organ, for there is no safe ground for the medical jurist, outside of the theory which is well supported, that unsoundness of the mind is due to disease of its organ.

According to Bucknell and Tuke, to whose excellent work I am greatly indebted, the earliest symptoms which mark the existence of this disease, are those due to a change in the natural and habitual character of the patient, which is generally gradual. He is more absorbed and reserved than usual, and under any provocation is unreasonably irritated. If formerly of a lively disposition and given to the pursuit of pleasure, this change will be most marked, or if formerly circumspect and correct in his deportment, he will commit excesses and carry them to such extremes as to attract the attention of his friends. He becomes suspicious and liable to attribute false motives to those with whom he is connected, and will cast ungenerous reflections on his nearest relatives without any foundation in fact. And as the disease becomes more advanced his acquaintances become conscious that he is greatly changed, without probably supposing him to be insane. He desires to be alone and becomes a victim to most gloomy forebodings. He finds himself susceptible to the slightest mental emotion; loses his sleep; his appetite fails; is conscious of uneasiness about his head; a sense of fulness and dull aching pain, and is distressed by impulses and tendencies which his reason and his moral sense both rebuke. His moral faculties at last are in more or less complete abeyance, and in the case of the pyromaniac, an incendiary act is committed without any reason, or if any motive be assigned, it is of so trivial a character as in no degree to justify the act. If not detected in his first attempt he will continue his depredation against friend and foe alike, wherever his fancy may lead him. Up to this point disorder of the circulation, as evinced by the pulse or of digestion, as indicated by the tongue, fæces or urine, may or may not be notably affected. Some serious physical disease, as epilepsy, will frequently now set in, or if not, and relief be not obtained, the patient as in monomanias in general, either becomes a maniac or passes into a state of dementia, followed by death. "The signs of reasoning monomania," says Esquirol,

"consist in a change in the habits, disposition and affections. The understanding is not essentially disturbed, since it assists in the acts of the insane person, and the patient is always ready to justify his sentiments and conduct. We distinguish three periods in it. In the first the disposition and habits are changed. In the second the affections are perverted, and at length in the third a maniacal excitement appears, or else a weakening of the faculties, more or less rapid, leads the monomaniac to dementia."

The cause of pyromania appears to be disease of the brain in consequence of abnormal development of the reproductive functions, and occurs generally about the period of puberty and early manhood. This was the fact in twenty cases collated by Kein and Platner, and a larger number of girls than boys were the subjects, and all the cases which I find recorded have occurred during early life, nearly half the patients being between the ages of sixteen and nineteen years. Marc observes that the period at which pyromania manifests itself corresponds nearly between the ages of twelve and twenty, and asserts that if an incendiary act has been committed by a person of this age in whom there exists any general symptoms indicative of irregular development, or of critical changes by which the attempt is being made to perfect the evolution of the reproductive system, is probably the result of disease. And he urges the necessity of ascertaining whether there is any disorder of the circulation, nervous system or cerebral functions.

As illustrating the foregoing description I will briefly relate a case which occurred in this vicinity, and will not therefore be without some interest. During the early part of last summer the village of Silver Creek, in an adjoining county, was thrown into a state of consternation and alarm by a rapid succession of fires set to the dwellings and barns of the inhabitants by some unknown incendiary. So great was their fear that they moved horses and cattle from the barns at night, and a watch was set in nearly every house. Watchmen also patrolled the streets at night. Suspicion finally pointed to Milton B. Wells, a native of the town, twenty-three years of age, unmarried, and of previous good character. He had been seen leaving one of the buildings which were burned, at night, and had been in one and at the time occupied the other of two buildings which were burned during the day, as a photograph gallery. There were other circumstances connected with the burning of these buildings which I need not mention that proved beyond a doubt that he was the incendiary. There was no reason why he should have set any of the fires, and particularly the building which he occupied, as there was no insurance on his goods which were destroyed. He was arrested and indicted for setting three of the fires. After having been some time in jail he was let to bail, and brought by his counsel, Josiah Cook, Esq., to Buffalo, at which time I saw him. He was also examined by Drs. Miner and Mixer, at the request of his counsel, for grounds of defence against the charge of arson. The history of the case showed that he in connection with another young man had for a long time been much addicted to onanism, and so well known was this to several persons in the village that they were often derided for their crime against nature. As a result of this practice he had been for a year or more under the care of Dr. Burwell of this eity for spermatorrhœa. Previous to his arrest his friends had noticed a gradual change in his character and habits, for which they could not account. Among other excesses he had at times taken to the free use of alcoholic liquor, and this by some was considered the cause of his incendiary acts. At the time of our examination he was reserved and melancholy, refusing to engage in extended conversation, which was unnatural in him, but would answer questions correctly, and was much given to reading. His general condition did not indicate any notable disorder of circulation or digestion, though he was quite anæmic, and his appearance was that of one suffering from some chronic disease. He was taken to a Western State and in three months brought before the court sitting at Mayville, Chautauqua county, for trial for arson. We were present and were called upon by the court to again examine the prisoner in connection with several other physicians as to his unsoundness of mind, which was set up as a defence by his counsel. His condition was greatly changed from what it was three months before. His pulse was 150, his head hot and injected, he recognized no one, could be made to comprehend nothing, was constantly muttering to himself, and his attention rivited to the floor, all the faculties of his mind in fact being entirely gone. As the law forbids the trial of persons of unsound mind the court upon these facts being presented to it dismissed the case, and restored the prisoner to his friends. He was immediately placed under medical care, but when last heard from his condition was not improved.

It will be observed that this is a typical case of pyromania in cause, course and result, as described by alienist physicians. His insanity was undoubtedly due to excitement and abnormal development of the reproductive organs as the result of excessive masturbation and consequent spermatorrhœa, as these are admitted by all writers to be sufficient causes to produce under certain circumstances this or almost any form of insanity. It was not hereditary, for there had never been a case of insanity in the relatives of either father or mother. Nor was it the result of intemperance, for he had never been addicted to the use of alcohol to excess, or even moderately, until a short time before the fires; and this was but a part of the change in his habits which had been going on for some time, and so gradual had been the change from health to insanity, that his physician, who was also a near neighbor, under whose care he had grown from infancy to early manhood, did not recognize the disease, but was his most active prosecutor for what he supposed to be malicious arson. There was not at any time maniacal excitement or delusion of any kind, and he stoutly denied from the first setting any of the fires which had occurred.

In this description I have purposely avoided allusion to instances of arson by persons of unsound mind occurring in more advanced age. It is possible that some such might be considered true cases of pyromania, but they have generally been maniacs, and cannot therefore be considered subjects of this disease. The majority of alienist writers have seemed to be desirous of placing this among the diseases of early life, on account of its occurring so uniformly at the period at which the reproductive organs are becoming active members of the system, and the frequency of cases of arson by young persons in whom disease of these organs has been discovered. While on the other hand some others, including the medical council of Berlin, have included all cases of this kind under the general head of destructive mania, instead of constituting it a distinct form of mental disease. Enough facts it would seem have been observed to plainly establish it as a distinct disease, and the difference of opinions by those of high authority on psychological medicine only shows how little is known as to the essential nature of insanity.

It would be a source of pleasure and profit could I now go on and accurately relate the changes which take place in the brain, if it is an organic disease, or what particular function is deranged, and its cause, or it is a functional disorder, but in the present state of our knowledge of mental disease this is impossible. It is surmised by some that the affection is in its commencement caused by exhaustion of nervous force, resulting from excitement of the reproductive organs, producing derangement of the functions of the brain, and according to the law which holds good in all organs, derangement of function produces congestion of that organ, then stasis, inflammation and its consequences. But this conjecture, even allowing it to be true, throws no light on the connection between disordered sexual functions and that state of mind which impels its victim to commit arson without a cause.

Bucknell observes, that "if our knowledge were complete of the cerebral organization we should from any morbid state of the cerebral capillaries and their contents be able to predict the anomalies of mental function which would result therefrom. To this end we should require to possess a knowledge of the state of the cells upon which morbid conditions of the circulation have to act, and herein lies the great difficulty of pathological science, that these minute but all-important constituents of the organization refuse to yield their secrets."

We must, therefore, wait until the relations of mind to brain matter in health and disease are better understood, and as nothing is positively known on these points, I will not longer tire your patience by reciting conjectures and unfounded theories, but will close, thanking you for your indulgence and kind attention.

---

A coroner's inquiry has taken place in the Wrexham district in consequence of the death of a patient under chloroform for an operation for fistula. The evidence of the two medical men, Messrs. Griffith and Jones, who were jointly concerned in the operation, most completely satisfied the jury that no blame whatever attached to them, the verdict being that "the deceased died from the effects of chloroform properly administered."—*London Lancet.*

